# New applications of Schrödinger type inequalities in the Schrödingerean Hardy space

**DOI:** 10.1186/s13660-017-1577-7

**Published:** 2017-12-13

**Authors:** Yong Lu, Liang Kou, Jianguo Sun, Guodong Zhao, Wenshan Wang, Qilong Han

**Affiliations:** 1 College of Power and Energy Engineering, Harbin Engineering University, Harbin, 150001 China; 2 Department of Computer Science and Technology, Harbin Engineering University, Harbin, 150001 China

**Keywords:** Schrödinger type inequality, Schrödingerean Hardy space, upper half-plane

## Abstract

As new applications of Schrödinger type inequalities obtained by Jiang (J. Inequal. Appl. 2016: Article ID 247, 2016) in the Schrödingerean Hardy space, we not only obtain the representation of Schrödingerean harmonic functions but also give a sufficient and necessary condition between the Schrödingerean distributional function and its derivative in the Schrödingerean Hardy space.

## Introduction

The Schrödingerean Hardy spaces $H^{p}(\mathbb{C_{+}})$ ($1 < p < \infty $) are defined to consist of those functions *f*, Schrödingerean holomorphic in the upper half-plane $\mathbb{C}_{+} =\{ z=x+iy:y>0\}$ with the property that $M_{p}(f,y)$ is uniformly bounded for $y > 0$, where

$$M_{p}(f,y)= \biggl( \int_{-\infty}^{+\infty} \bigl\vert f(x+iy) \bigr\vert ^{p}\,dx \biggr)^{\frac{1}{p}}. $$

Since $|f|^{p}$ is Schrödingerean subharmonic for $f \in H^{p}(\mathbb {C_{+}})$ with respect to $\mathit{Sch}_{a}$, the function $M_{p}(f,y)$ decreases in $(0,\infty)$,

$$\Vert f \Vert _{H^{p}(\mathbb{C_{+}})}=\sup\bigl\{ M_{p}(f,y):0< y< \infty\bigr\} =\lim_{y\to0}M_{p}(f,y). $$

If $f(x)$ is the non-tangential boundary limits of the Schrödingerean function $f \in H^{p}(\mathbb{C_{+}})$, then $f(x) \in L^{p}(\mathbb{R})$ and

$$\Vert f \Vert _{p}= \biggl( \int_{-\infty}^{+\infty} \bigl\vert f(x) \bigr\vert ^{p}\,dx \biggr)^{\frac{1}{p}}= \Vert f \Vert _{H^{p}(\mathbb{C_{+}})}. $$

A function $\phi(t)$ defined on $\mathbb{R}$ belongs to the space $\mathcal{D}_{L^{p}}$, $1 < p < \infty$, iff

1. $\phi(t)\in\mathcal{C}^{\infty}$;
2. $\phi^{(k)}(t)\in L^{p}$ for all $k\in\mathbb{N}$, where $\mathbb {N}$ is the set of nonnegative integers.

The space $\mathcal{D}$ consists of infinitely differentiable complex-valued functions defined on $\mathbb{R}$. In the sequel, for $1 < p < \infty$, we will write

$$p'=\frac{p}{p-1} $$

and denote by $\mathcal{D}'_{L^{p}}$ the dual of the space $\mathcal{D}_{L^{p^{\prime}}}$, that is, $\mathcal{D}\prime_{L^{p}}=(\mathcal{D}_{L^{p^{\prime}}})^{\prime}$. We also denote by $D^{\prime}$ the dual of the space *D*. So we can get $D\subseteq\mathcal{D}_{L^{p}}$ and $\mathcal{D}'_{L^{p}}\subseteq D^{\prime}$.

### Definition 1.1

(see [2])

Let $f \in\mathcal{D}^{\prime}$. An analytic representation of *f* is any function $F(z)$ defined and analytic on the complement of the support of *f* such that for all test functions $\phi\in\mathcal{D}$,

$$\lim_{y\to0^{+}} \int_{-\infty}^{+\infty} \bigl[F(x+iy)-F(x-iy) \bigr]\phi(x)\,dx= \bigl\langle f(x),\phi(x) \bigr\rangle . $$

### Definition 1.2

Let $f \in\mathcal{D}'_{L^{p}}$ ($1 < p <\infty$) and assume that *Df* is the operator of distributional differentiation defined on $\mathcal {D}'_{L^{p}}$ by

$$\langle Df,\phi \rangle= \langle f,-D\phi \rangle $$

for all $\phi\in\mathcal{D}_{L^{p^{\prime}}}$.

Then

$$Df\in\mathcal{D}^{\prime}_{L^{p}}. $$

Since $f\in\mathcal{D}^{\prime}_{L^{p}}$,$D\varphi\in D_{L^{p^{\prime }}}$, *Df* defined as above is a functional on $D_{L^{p^{\prime}}}$. Linearity of *Df* is trivial. Assume that $\{\varphi_{v}\}\to\varphi$ in $D_{L^{p^{\prime}}}$. Then

$$\langle Df,\varphi_{v} \rangle= \langle f,-D\varphi _{v} \rangle\to \langle f,-D\varphi \rangle= \langle Df,\varphi \rangle. $$

## Main results

In 2016, Jiang (see [1]) proved Schrödinger type inequalities for stabilization of discrete linear systems associated with the stationary Schrödinger operator. As applications, Jiang and Uso (see [3]) obtained boundary behaviors for linear systems of subsolutions of the stationary Schrödinger equation. Almost at the same time, Huang (see [4]) considered a new type of minimal thinness with respect to the stationary Schrödinger operator. As an application, Huang and Ychussie (see [5]) solved the Dirichlet-Sch problems on smooth cones with slow-growth continuous data. Recently, Lü and Ülker (see [6]) gave the existence of weak solutions for two-point boundary value problems of the Schrödingerean predator-prey system. Motivated by their results, by using Schrödinger type inequalities proved by Jiang (see [1]), we obtain the integral representation of Schrödingerean harmonic functions in the Schrödingerean Hardy space.

### Theorem 2.1

*Suppose that* $1< p<\infty$ *and* $f\in\mathcal{D}^{\prime}_{L^{p}}$. *Then*

$$F(Z)=\frac{1}{2\pi i}\biggl\langle f(t),\frac{1}{t-z}\biggr\rangle $$

*is one of the analytic representations of* *f*, *which satisfies*

$$\sup_{-\infty< x< \infty,y\ge\delta>0}\big\| F(x+iy)\big\| =A_{\delta}< \infty $$

*and*

$$\sup_{-\infty< x< \infty}\big\| F(x+iy)\big\| =O\bigl(y^{-\frac{1}{p}}\bigr), $$

*where* $y\to\infty$.

*There exist functions* $F_{k}(z)\in H^{p}(\mathbb{C}_{+})$, *so that*

$$F(z)=\sum_{k=1}^{r}\frac{\partial^{k-1}}{\partial z^{k-1}}F_{k}(z) $$

*and*

$$F^{(j)}(z)=\sum_{k=1}^{r} \frac{\partial^{k+j-1}}{\partial z^{k+j-1}}F_{k}(z), $$

*where* *r* *and* *j* *are nonnegative integers*.

### Theorem 2.2

*If* $1< p<\infty$, $F_{k}\in H^{p}(\mathbb{C}_{+})$ *and*

$$F(z)=\sum_{k=1}^{r}\frac{\partial^{k-1}}{\partial z^{k-1}}F_{k}(z), $$

*then there exists a distributional function* $f(x)\in\mathcal{D}^{\prime}_{L^{p}}$ *such that* $F(z)$ *is one of analytic representations of* $f(x)$.

### Corollary 2.3

*If* $1< p<\infty$ *and* $f(x)\in\mathcal {D}^{\prime}_{L^{p}}$, *then*

$$F(Z)=\frac{1}{2\pi i}\biggl\langle f(t),\frac{1}{t-z}\biggr\rangle $$

*satisfies*

$$\sup_{-\infty< x< \infty,y\ge\delta>0}\big\| F(x+iy)\big\| =A_{\delta}< \infty $$

*and*

$$\sup_{-\infty< x< \infty}\big\| F(x+iy)\big\| =O\bigl(y^{-\frac{1}{p}}\bigr), $$

*where* $y\to\infty$.

*There exist functions* $F_{k}(z)$ *in* $H^{p}(\mathbb{C}_{+})$ *so that*

$$F(z)=\sum_{k=1}^{r}\frac{\partial^{k+j-1}}{\partial z^{k+j-1}}F_{k}(z), $$

*where* $j>1$, *r*, *j* *are nonnegative integers*.

## Lemmas

In this section we need the following lemmas.

### Lemma 3.1

(see [7, 8])

*If* $1< p<\infty$, $u(t)\in L^{p}(\mathbb{R})$ *and the function* $G(u)(t)$ *is defined as follows*

$$G(u) (t)=\frac{1}{2\pi i} \int_{-\infty}^{\infty}\frac{u(t)}{t-z}\,dt, $$

*then*

$$G(u) (t)\in H^{p}(\mathbb{C}_{+}). $$

### Lemma 3.2

(see [1])

*Let* $F(z)$ *be an analytic complex*-*valued function of the complex variable* $z = x + iy$ *in the open upper half*-*plane satisfying*

1. *for fixed* $y>0$, $p\prime=\frac{p}{p+1}$, $1< p<\infty$, $F(x+iy)\in L^{p}$;
2. $$\lim_{y\to0^{+}}F(x+iy)=f^{+}(x) $$
  *in* $\mathcal{D}^{\prime}_{L^{p}}$ (*weakly*)
  $$\sup_{-\infty< x< \infty}\big\| F(x+iy)\big\| \to O, $$
  *where* $y\to\infty$ *and*
  $$\sup_{-\infty< x< \infty,y\ge\delta>0}\big\| F(x+iy)\big\| =A_{\delta}< \infty. $$

*Then*

$$F(z)=\frac{1}{2\pi i}\biggl\langle f^{+}(t),\frac{1}{t-z}\biggr\rangle , $$

*where* $\operatorname{Im}z>0$.

## Proofs of the main results

### Proof of Theorem 2.1

In view of the structure formula in [3], for $f\in\mathcal{D}^{\prime}_{L^{p}}$,

$$F(z)=\frac{1}{2\pi i}\biggl\langle f^{+}(t),\frac{1}{t-z}\biggr\rangle , $$

there exists a nonnegative integer *r* and $f_{k}\in L_{p}$ such that

$$\begin{aligned} F(z)&=\frac{1}{2\pi i}\sum_{k=1}^{r} \int_{\mathbb{R}} f_{k}(t) \biggl(-\frac {\partial}{\partial t} \biggr)^{(k-1)}\biggl(\frac{1}{t-z}\biggr)\,dt \\ &=\frac{1}{2\pi i}\sum_{k=1}^{r} \int_{\mathbb{R}} f_{k}(t) (-1)^{k-1} \frac{(k-1)!}{(t-z)^{k}}\,dt.\end{aligned} $$

So

$$\big|F(x+iy)\big|\le\frac{1}{2\pi}\sum_{k=1}^{r} \int_{\mathbb{R}} \big|f_{k}(t)\big|\frac{(k-1)!}{|t-z|^{k}}\,dt. $$

By using Holder’s inequality, we have

$$\big|F(x+iy)\big|\le\frac{1}{2\pi}\sum_{k=1}^{r}(k-1)! \biggl( \int_{\mathbb{R}} \big|f_{k}(t)\big|^{p} \,dt \biggr)^{\frac{1}{p}} \biggl( \int_{R} \frac {1}{|t-z|^{kp^{\prime}}}\,dt \biggr)^{\frac{1}{p^{\prime}}}. $$

Define

$$I= \int_{R} \frac{1}{|t-z|^{kp^{\prime}}}\,dt. $$

So

$$\begin{aligned} I&= \int_{\mathbb{R}} \frac{1}{[(t-x)^{2}+y^{2}]^{\frac{kp^{\prime}}{2}}}\,dt \\ &= \int_{\mathbb{R}} \frac{1}{y^{kp\prime}[(\frac{t}{y})^{2}+1]^{\frac {kp^{\prime}}{2}}}\,dt \\ &=\frac{1}{y^{kp\prime-1}} \int_{\mathbb{R}}\frac{1}{(1+t^{2})^{\frac {kp^{\prime}}{y}}}\,dt.\end{aligned} $$

Further, $k>1$ and $p^{\prime}>1$ imply that $kp\prime> 1$ for $y \ge \delta> 0$, there exists a constant *C* satisfying

$$I\le\frac{C}{\delta^{kp\prime-1}}< \infty. $$

Since $f_{k} \in L^{p}$, there exists a constant *M* such that

$$\sup_{-\infty< x< \infty,y\ge\delta>0}\big\| F(x+iy)\big\| =A_{\delta}< \infty, $$

where

$$\begin{gathered} A_{\delta}=\frac{1}{2\pi}\sum_{k=1}^{r}(k-1)! \frac{MC^{\frac {1}{p^{\prime}}}}{\frac{k\prime-1}{\delta p\prime}}, \\\big|y^{\frac{1}{p}}F(x+yi)\big|\le\sum_{k=1}^{r}(k-1)! \|f_{k}\|_{L}^{p}\frac {1}{y^{kp\prime-1-\frac{1}{p}}} \int_{\mathbb{R}}\frac {1}{(1+t^{2})^{\frac{kp^{\prime}}{y}}}\,dt\end{gathered} $$

and

$$kp\prime-1-\frac{1}{p}=p^{2}(1-k)-1< 0. $$

So

$$\lim_{y\to\infty}\sup_{-\infty< x< \infty}\big|F(x+yi)\big|=O \bigl(y^{-\frac{1}{p}}\bigr). $$

In view of the structure formula (see [9])

$$\begin{aligned} F(z)&=\frac{1}{2\pi i}\sum_{k=1}^{r} \int_{\mathbb{R}} f_{k}(t) \biggl(-\frac {\partial}{\partial t} \biggr)^{(k-1)}\biggl(\frac{1}{t-z}\biggr)\,dt \\ &=\frac{1}{2\pi i}\sum_{k=1}^{r} \int_{\mathbb{R}} f_{k}(t) \biggl(\frac {\partial}{\partial z} \biggr)^{(k-1)}\biggl(\frac{1}{t-z}\biggr)\,dt \\ &=\sum_{k=1}^{r}\biggl(\frac{\partial}{\partial z} \biggr)^{(k-1)}\frac{1}{2\pi i} \int_{\mathbb{R}} \frac{f_{k}(t)}{t-z}\,dt \\ &=\sum_{k=1}^{r}\biggl(\frac{\partial}{\partial z} \biggr)^{(k-1)}F_{k}(z),\end{aligned} $$

where

$$F_{k}(z)=\frac{1}{2\pi i} \int_{\mathbb{R}} \frac{f_{k}(t)}{t-z}\,dt. $$

According to Lemma 3.1, we know that $F_{k}(z)\in H^{p}(C_{+})$, which gives that

$$F^{(j)}(z)=\sum_{k=1}^{r}\biggl( \frac{\partial}{\partial z}\biggr)^{(k+j-1)}F_{k}(z), $$

where *j* is a nonnegative integer.

### Proof of Theorem 2.2

Since $F_{k}(z)\in H^{p}(C_{+})$, there exist functions $f_{k}(t)\in L^{p}$, where $f_{k}$ is the non-tangential limit of $F(z)$, where $F_{k}(x+iy)\in L^{p}$ for fixed *y*.

Since $D_{L^{p^{\prime}}}\in L^{p^{\prime}}$, we see that $f_{k}(t)\in D^{\prime}_{L^{p}}$. By using the property of a subharmonic function, we get

$$\begin{aligned} \big|F_{k}(x+iy)\big|^{p}&\leq\frac{1}{\pi y^{2}} \int_{D(x+iy,y)}\big|F_{k}(\xi+i\eta )\big|^{p}\,d\lambda \\ &\leq\frac{1}{\pi y^{2}} \int_{x-y}^{x+y} \int_{0}^{2y}\big|F_{k}(\xi+i\eta )\big|^{p}\,d\eta \,d\zeta \\ &\leq\frac{2}{\pi y}\|F_{k}\|_{H^{p}}^{p}.\end{aligned} $$

So

$$\big|F_{k}(x+iy)\big|\le\biggl(\frac{2}{\pi y}\|F_{k} \|_{H^{p}}^{p}\biggr)^{\frac {1}{p}}=y^{\frac{1}{p}} \biggl(\frac{2}{\pi}\|F_{k}\|_{H^{p}}^{p} \biggr)^{\frac{1}{p}}, $$

which gives that

$$F_{k}(x+iy)=O\biggl(\frac{1}{y\frac{1}{p}}\biggr), $$

where $y>0$ and

$$\sup_{-\infty< x< \infty,y\ge\delta>0}\big\| F(x+iy)\big\| \le\frac{2}{\pi \delta}\|F_{k} \|_{H^{p}}^{p}=A_{\delta}< \infty. $$

According to Lemma 3.2, we know that $F_{k}(z)$ can be written as

$$F_{k}(x)=\frac{1}{2\pi i}\biggl\langle f_{k}(t),\frac{1}{t-z}\biggr\rangle . $$

So

$$\begin{aligned} F(z)&=\sum_{k=1}^{r}\biggl( \frac{\partial}{\partial z}\biggr)^{k-1}F_{k}(z) \\ &=\sum_{k=1}^{r}\biggl(\frac{\partial}{\partial z} \biggr)^{k-1}\frac{1}{2\pi i} \biggl\langle f_{k}(t), \frac{1}{t-z} \biggr\rangle \\ &=\frac{1}{2\pi i}\sum_{k=1}^{r} \biggl\langle f_{k}(t),\biggl(\frac {\partial}{\partial z}\biggr)^{k-1} \frac{1}{t-z} \biggr\rangle \\ &=\frac{1}{2\pi i}\sum_{k=1}^{r} \biggl\langle D^{(k-1)}f_{k}(t),\frac {1}{t-z} \biggr\rangle \\ &=\frac{1}{2\pi i} \Biggl\langle \sum_{k=1}^{r} D^{(k-1)}f_{k}(t),\frac{1}{t-z} \Biggr\rangle ,\end{aligned} $$

which gives that

$$\begin{aligned} F(z)&=\sum_{k=1}^{r}\biggl( \frac{\partial}{\partial z}\biggr)^{k-1}F_{k}(z) \\ &=\sum_{k=1}^{r}\biggl(\frac{\partial}{\partial z} \biggr)^{k-1}\frac{1}{2\pi i} \biggl\langle f_{k}(t), \frac{1}{t-z} \biggr\rangle \\ &=\frac{1}{2\pi i}\sum_{k=1}^{r} \biggl\langle f_{k}(t),\biggl(\frac {\partial}{\partial z}\biggr)^{k-1} \frac{1}{t-z} \biggr\rangle \\ &=\frac{1}{2\pi i}\sum_{k=1}^{r} \biggl\langle D^{(k-1)}f_{k}(t),\frac {1}{t-z} \biggr\rangle \\ &=\frac{1}{2\pi i} \Biggl\langle \sum_{k=1}^{r} D^{(k-1)}f_{k}(t),\frac {1}{t-z} \Biggr\rangle .\end{aligned} $$

Let

$$f(x)=\sum_{k=1}^{r} D^{(k-1)}f_{k}(t), $$

where $f(x)\in D^{\prime}_{L^{p}}$, which implies that $F(z)$ is one of the analytic representations of $f(x)$.

### Proof of Corollary 2.3

In view of the structure formula (see [9])

$$\begin{aligned} F(z)&=\frac{1}{2\pi i} \biggl\langle f(t),\frac{1}{(t-z)^{j}} \biggr\rangle \\ &=\frac{1}{2\pi i}\sum_{k=1}^{r} \int_{\mathbb{R}} f_{k}(t) \biggl(-\frac {\partial}{\partial t} \biggr)^{(k-1)}\biggl(\frac{1}{(t-z)^{j}}\biggr)\,dt \\ &=\frac{1}{2\pi i}\sum_{k=1}^{r} \int_{\mathbb{R}} f_{k}(t)\frac {(k+j-2)!}{(j-1)!(t-z)^{k+j-1}}\,dt.\end{aligned} $$

Similar to the proof of Theorem 2.1, we can see the corollary holds.

## Conclusions

In this paper, we not only obtained the representation of Schrödingerean harmonic functions but also gave a sufficient and necessary condition between the Schrödingerean distributional function and its derivative in the Schrödingerean Hardy space.
